# A Hierarchical Ensemble Deep Learning Activity Recognition Approach with Wearable Sensors Based on Focal Loss

**DOI:** 10.3390/ijerph191811706

**Published:** 2022-09-16

**Authors:** Ting Zhao, Haibao Chen, Yuchen Bai, Yuyan Zhao, Shenghui Zhao

**Affiliations:** School of Computer and Information Engineering, Chuzhou University, Chuzhou 239000, China

**Keywords:** activity recognition, deep learning, wearable sensors

## Abstract

Abnormal activity in daily life is a relatively common symptom of chronic diseases, such as dementia. There will probably be a variety of repetitive activities in dementia patients’ daily life, such as repeated handling of objects and repeated packing of clothes. It is particularly important to recognize the daily activities of the elderly, which can be further used to predict and monitor chronic diseases. In this paper, we propose a hierarchical ensemble deep learning activity recognition approach with wearable sensors based on focal loss. Seven basic everyday life activities including cooking, keyboarding, reading, brushing teeth, washing one’s face, washing dishes and writing are considered in order to show its performance. Based on hold-out cross-validation results on a dataset collected from elderly volunteers, the average accuracy, precision, recall and F1-score of our approach are 98.69%, 98.05%, 98.01% and 97.99%, respectively, in identifying the activities of daily life for the elderly.

## 1. Introduction

Elderly people may suffer from the consequences of dementia. Dementia may cause a decrease in the ability to speak, write and perform complex functional tasks, such as preparing a meal.

Most common types of dementia can be identified by a change in daily activities such as sleep disturbances, difficulty walking and an inability to complete tasks. Such changes can provide key information about the memory, mobility and cognition of a person. For instance, an inhabitant suffering from Alzheimer’s may forget his lunch, or go to the toilet frequently. The best markers of cognitive decline may not necessarily be detected based on a person’s activities at any single point in time, but rather by monitoring the trend over time and the variability of change in a duration. Therefore, it is important to recognize and monitor the activities that can better detect the health status of the elderly. In recent years, with the development of microelectronics and low-power wireless technology, the cost of wearable sensor devices has been greatly reduced. In addition, wearable devices have the advantages of small size, low power consumption, easy integration, and high recognition accuracy of human activities. Wearable devices can collect human activity data, which provides the possibility for activity recognition without affecting the comfort of daily activities.

Early researches focused on using different machine learning models to recognize users’ activity, such as the HMM [1], naive Bayes classifier [2] and decision tree [3]. However, manual feature selection not only requires a wealth of medical knowledge, but the process requires trial and error, which consumes much time and effort. These will lead to a low recognition accuracy. Recently, deep learning has been successfully applied in image classification [4] and image description [5]. For example, researchers have implemented a wearable sensor activity recognition system based on deep learning [6], which extracts the hidden features of sensor data automatically, captures complex activity details and improves the accuracy and robustness of activity recognition.

However, the numbers of different human activity-sensing data are often unbalanced. Some categories have more samples than others. For example, typing or writing activities have more samples than washing dishes. In this case, the trained model will be biased towards one category, which has more data. Thus, it will cause the minority categories to be misclassified, and even treat them as noise. In other words, because each epoch’s categories are unbalanced, the model is more and more accurate in classifying the samples of the majority category; meanwhile the recognition on the minority category is getting worse. So, the accuracy rate cannot be used as the key indicator for evaluating the model.

In addition, human beings’ daily activities are complex. On the one hand, the distribution of activity data in the same category is different because of a person’s different exercise habits at different times. On the other hand, the sensor has various heterogeneities that make the sensitive information of human activities unable to keep synchronized after the fusion of multiple sensor data. Furthermore, a person’s different categories of activities have similarities. To sum up, the traditional single model cannot guarantee accurate recognition performance.

To address these problems, this paper designs and proposes a hierarchical ensemble deep learning activity recognition scheme. This scheme provides wearable sensors to patients for both wrists, and a variety of human daily activity data are collected by the sensors. Then, after data preprocessing and analyzing, a hierarchical ensemble deep learning activity recognition scheme based on focal loss is designed for the imbalance of dataset categories, and testing of the trained model. The contributions of this paper can be summarized in the following aspects:

(1) This paper analyzes the sensitivity of a wearable inertial sensor on the wrist to human activity. For the same sensor, the data generated by different activities are quite different, and for different sensors, the data generated by the same action are relatively different.
(2) In view of the complexity and imbalance of human daily activity data, after preprocessing the data, this paper proposes a deep hierarchical ensemble learning model based on focal loss, and designs an elderly daily activity recognition system based on wearable sensors.
(3) This paper employs real experimental data to evaluate the performance of the proposed method and compares it with some state-of-the-art methods in the literature. Furthermore, this paper evaluates the impact of some key hyperparameters using experimental data.

This paper is divided into five sections. Section 1 is the introduction. Section 2 includes the previous studies that have been carried out so far. The proposed scheme is examined in Section 3. The experimental results and analysis are described in Section 4, and the conclusion is discussed in Section 5.

## 2. Related Work

With the development of a wireless sensor network and the gradual popularization of wearable sensors, it is worthwhile to build activity recognition systems based on wearable wireless sensors. Activity recognition systems have been widely used and scientifically studied by many scholars and institutions. Researchers attach sensors to the human body’s key parts, and use acceleration sensors to measure the acceleration data of each part continuously. After that, these data are sent to a base station through the Bluetooth wireless network. Usually, the base station is a sensor that is connected to a computer or mobile phone. Therefore, these sensor data provide effective support for in-depth research on activity recognition.

In the early days, different machine learning methods were mainly used to identify wearable-based human activities. The common methods include: KNN, HMM, SVM, RF, XGBoost, etc. For example, Lee and Cho [7] used a hierarchical hidden Markov model to identify five types of activities, including standing, walking, running, going upstairs and going downstairs. Data for these activities were acquired via a three-axis accelerometer on a smartphone. Kwapisz et al. [8] placed smartphones in the front pockets of users’ pants and collected 29 users’ accelerometer daily activities data, including walking, jogging, stair climbing, sitting and standing. They used these data to extract 6 different features and used 4 classifiers for identification. The recognition rate reached more than 90%. Sun et al. [9] proposed a sports activity recognition scheme based on SVM, which placed smartphones in 6 different pockets, collected 7 sports activity data, and trained an SVM activity recognition classifier. The total F-score reached 94.8% given the pocket position.

The process of activity recognition needs large amounts of domain knowledge and extracted features with trial and error. This process represents a major expenditure of time and effort. In recent years, with the development and application of deep learning technology [10], there has been a lot of related work in the field of activity recognition.

Jiang et al. [11] constructed an activity feature map through the signal sequence of the accelerometer and the gyroscope. Then they used the deep CNN network to learn the optimal features of multiple dimensions automatically, and achieved a better recognition effect. Ronao et al. [12] used time-series sensor data to predict activities, confirming the effectiveness of 2D-CNN for activity recognition. Ravi et al. [13] collected activity data with low-power wearable devices. They processed the time series through short-time Fourier transform (STFT) spectrograms, then designed a deep learning-based human activity recognition architecture, and finally achieved accurate real-time classification. Amroun et al. [14] collected four types of activity data, including standing, sitting, lying down and walking, to extract the best feature descriptors of activities, and identified human activities through the CNN model, with a recognition accuracy rate of over 98%. Reference [15] designed a LSTM network, then performed experimental evaluation on three standard benchmark (Opportunity, PAMAP2, Skoda) datasets, and finally achieved better recognition results. The above systems all used a single model for activity recognition. However, existing studies have shown that the integrated model has better performance [16].

To learn hierarchical features, Ref. [17] adopted RBMs and multi-layer RBMs are used to capture local and multimodal features for human action recognition. Ordóñez et al. [18] used wearable sensors to build convolutional and recurrent network architectures to extract behavioral features automatically and improved system performance. Chen et al. [19] designed an integrated ELM algorithm based on smartphone sensors. The algorithm identified human activities such as walking, going upstairs, going downstairs, sitting, standing and walking, and the recognition accuracy reached 97.35%. Reference [20] proposed a lightweight and efficient integrated incremental learning activity recognition system based on the heterogeneous activity recognition datasets of multiple users and sensing devices. After model testing, the results showed a 35% improvement in accuracy.

To address the problem of unbalanced data categories, there are mainly two methods. On the one hand, a data-level method that operates on the training set and changes its class distribution. For example, the reference [21] simply replicated selected samples randomly from the minority class to solve the problem of data class imbalance, and the reference [22] adopted a clustering-based oversampling method. First, they clustered the dataset, then oversampled each cluster. On the other hand is a classifier (algorithmic)-level method that keeps the training dataset distribution and adjusts the training or inference algorithm. For example, to keep the sample classes balanced, OHEM [23] proposed an idea that selected more minority class samples in each mini-batch iteration. The reference [24] reduced the weight of the negative samples of the minority class in the training process by weighting the instances, focusing on the hard-to-classify and misclassified samples.

The comparison of related work is shown in Table 1. We can find that most of the related work mainly uses the data collected by the sensors in the smartphone for activity recognition. In contrast to existing work, we mainly focus on wearable sensor-based activity recognition at home.

Aiming at the complexity of human daily activities and the imbalance of data categories, this paper designs a human activity recognition architecture based on hierarchical ensemble learning that applies the focal loss algorithm to the system and improves the recognition effectively.

## 3. System Framework

**Figure 1:**
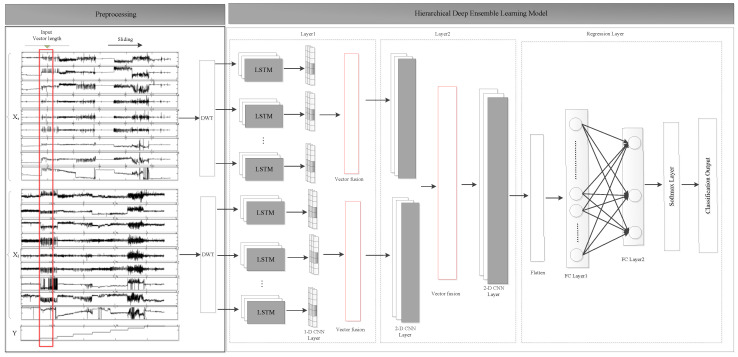
Activity recognition network architecture based on deep ensemble learning.

In order to identify the daily activities of the elderly, in this paper, wearable sensors were worn on both wrists of the volunteers to collect raw data, and for the class imbalanced dataset, an activity recognition network for the elderly based on hierarchical ensemble deep learning architecture was designed. The specific module design is shown in Figure 1.

In the activity recognition system, when the class samples are not balanced the trained model will be biased towards the class with more instances, resulting in the misclassification of the minority class samples. In this paper, the focal loss algorithm is applied to the activity recognition system, which can reduce the impact of sample imbalance.

### 3.1. Formal Description of Data

Before the dataset is inputted into the training model, the training data needs to be reconstructed into the data format required by the time series prediction model. For example, the size of the image input is fixed to h × w × c, where h, w, and c are the height, width and number of the images, respectively. In this section we describe in detail the pipeline for data preprocessing and the method for signal representation.

As shown in Figure 1, the sensor IMU signals at different body positions are synchronized with timestamps, and then, the signal sequence is sampled using a time sliding window with a width of T timestamps and the step size between the two windows is ∆*t*; after sampling, the dataset is represented as $D=\left\{\left[D_{1},y_{1}\right],\ldots ,\left[D_{n},y_{n}\right],\ldots ,\left[D_{N},y_{N}\right]\right\}$, and the nth data is represented as $D_{n}=\left[d_{n}^{1},d_{n}^{2},\ldots ,d_{n}^{s},\ldots d_{n}^{S}\right],\;n\epsilon \left\{1,\ldots ,N\right\}$, where *S* is the total number of IMU sensors at different body positions, $d_{n}^{s}$ represents the sample set of discrete time series IMU signals from the *s*th sensor, and $y_{n}$ is the activity class label. More specifically, $d_{n}^{s}=\left\{d_{n,1}^{s},d_{n,2}^{s},\ldots ,d_{n,t}^{s},\ldots ,d_{n,T}^{s}\right\}$ is a discrete-time data sequence over T timestamps, each element can be expressed as

(1) $$d_{n,t}^{s}=\left[\underset{a_{n,t}:acceleration}{\underbrace{a_{n,t}^{x},a_{n,t}^{y},a_{n,t}^{z}}},\underset{w_{n,t}:angular\;velocity\;}{\underbrace{w_{n,t}^{x},w_{n,t}^{y},w_{n,t}^{z}}},\underset{\;ag_{n,t}:angle\;}{\underbrace{ag_{n,t}^{x},ag_{n,t}^{y},ag_{n,t}^{z}}},\ldots \right]$$

where *a*, *g*, and *ag* represent the sensor readings of acceleration, angular velocity, and angle, respectively.

### 3.2. Wavelet Transform

In order to better represent the inertial signal, capture time and frequency information, we decompose the original signal into high-frequency components and low-frequency components, and obtain each layer of frequency signal information, because the human activity signals collected by wearable sensors are nonlinear and non-stationary. Therefore, it is very suitable to use the wavelet decomposition method [25] to analyze the signal.

Let the input signal be *x*, with the scale *j*, the wavelet coefficient $x,\psi_{j,k}$ and the scale coefficient $x,\phi_{j,k}$ can be obtained after decomposition, where $k=0,1,\ldots ,N_{j}-1$, that is to convolve the input signal with the given filters h and g at the same time

(2) $$h\left(t\right)=2^{-j/2}\psi \left(-2^{j}t\right)$$

(3) $$g\left(t\right)=2^{-j/2}\phi \left(-2^{-j}t\right)$$

Here, *ψ*(∙) represents the wavelet function, and *g*(∙) represents the scaling function, by discarding high-frequency components (details) and preserving low-frequency components to obtain a smooth output.

### 3.3. Hierarchical Ensemble Deep Learning Architecture

In order to extract the deep features of activities, we propose a novel hierarchical ensemble of neural networks. The architecture firstly extracts the features of each sensor data, considering that comprehensive analysis of correlations across each sensor data is essential for learning sensitivity features of activities. Hence, we extract features and learn the correlations across each sensor data through the fusion layer.

#### 3.3.1. Single-Channel Sensor Signal Feature Extraction

**Figure 2:**
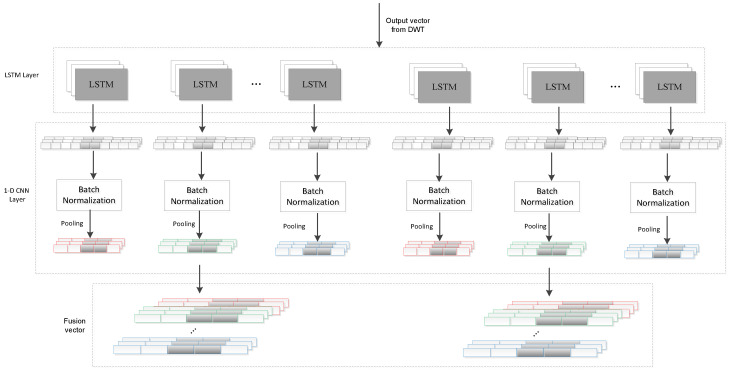
Single-channel sensor signal feature extraction.

By combining wave transform with the LSTM network in extracting the features of each sensing activity window, the time characteristics of each channel are acquired. Then, the 1D convolutional neural network (CNN) was used to extract local spatial features, as shown in Figure 2.

##### LSTM Layer

The cell status of LSTM can only be changed by a specific gate. A typical LSTM contains a forget gate, input gate, and output gate, which are represented by $f_{t}$, $i_{t}$,and $O_{t}$ respectively. Where, cell state, input and output are vectors represented by $C_{t}$, $x_{t}$ and $h_{t}$ respectively.

(4) $$f_{t}=\sigma \left(W_{f}h_{t-1}+U_{f}x_{t}+b_{f}\right)$$

(5) $$i_{t}=\sigma \left(W_{i}h_{t-1}+U_{i}x_{t}+b_{i}\right)$$

(6) $$a_{t}=\tanh\left(W_{c}h_{t-1}+U_{c}x_{t}+b_{c}\right)$$

(7) $$c_{t}=f_{t}*c_{t-1}+i_{t}*a_{t}$$

(8) $$o_{t}=\sigma \left(W_{o}h_{t-1}+U_{o}x_{t}+b_{o}\right)$$

(9) $$h_{t}=o_{t}*\tan h\left(c_{t}\right)$$

The forget gate determines whether to delete the contents of the cell state. The input gate decides what information will be stored in the memory cell. The forget gate and input gate determine the contents of the new cell state. The input of the output gate is determined by the previous output vector $h_{t-1}$ and the current input vector $x_{t}$. Where $a_{t}$ represents the information to be input to the memory, $W_{f}$, $U_{f}$,$\;W_{i}$, $U_{i}$, $W_{c}$, $U_{c}$, $W_{o}$, $U_{o}$ represent the weight, and $b_{f},b_{i},b_{c},b_{o}$ represent the offset, $\sigma \left(x\right)={\left(1+e^{-x}\right)}^{-1}$.

##### 1D-CNN Layer

With a one-dimensional sensor signal, a 1D kernel is used in a temporal convolution. A kernel can be viewed as a filter or a feature detector in the 1D domain. The method of extracting the feature map by using the one-dimensional convolution operation is as follows:

(10) $$x_{j}^{l+1}\left(\tau \right)=\sigma \left(b_{j}^{l}+\overset{F^{l}}{\underset{f=1}{\sum }}K_{jf}^{l}\left(\tau \right)*x_{f}^{l}\left(\tau \right)\right)=\sigma \left(b_{j}^{l}+\overset{F^{l}}{\underset{f=1}{\sum }}\left[\overset{p^{l}}{\underset{p=1}{\sum }}K_{jf}^{l}\left(p\right)x_{f}^{l}\left(\tau -p\right)\right]\right)$$

where, $x_{j}^{l}$ represents the *j*th feature map of *l* layer. σ is the nonlinear activation function. $F^{l}$ represents the number of feature map at the l layer. $K_{jf}^{l}$ is the kernel convolved over feature map f in layer l to create the feature map j in layer l+1. $p^{l}$ represents the length of the convolution kernel at the l layer, and $b^{l}$ is the offset vector.

In the process of model training, in order to reduce the internal covariate shift, a batch normalization layer is set behind each activation layer [26]. With a one-dimensional signal of kth sensing channel, we get $x^{k}$ output through 1D-CNN. In the small batch processing, there are γ activation values, which can be represents as $B=\left\{x_{1\ldots \gamma }^{k}\right\}$, by batch normalization layer. Thus, the output is defined by:

(11) $${\hat{x}}^{k}=BN\left\{x_{1\ldots \gamma }^{k}\right\}$$

where, ${\hat{x}}^{k}$ represents the output through a batch normalization layer of 1D-CNN layer in *k*th sensing channel. We set the max-pooling layer of size 2 for the data flows, which is the output of batch normalization layer. $x_{j}^{l}$ as the input to the pooling layer, represents *j*th feature map of the lth layer.

(12) $$x_{j}^{l+1}=MaxPooling\left(x_{j}^{l}\right)$$

##### Fusion Layer

In order to extract the correlation feature between each sensor channel, the output vectors of each channel in the fusion layer are combined, as shown in the following formula, where $C^{i}$ represents the splice result of the *i*th sensor vector, then

(13) $$C^{i}=\left[x_{1}^{i}⊙x_{2}^{i}\ldots x_{n}^{i}\right]$$

where $x_{k}^{i}$ represents the output of the *k*th channel of the *i*th sensor that flows through 1D-CNN layer, and ⊙ represents the splicing of vectors.

#### 3.3.2. Feature Fusion Extraction of Multi-Sensor Signals

**Figure 3:**
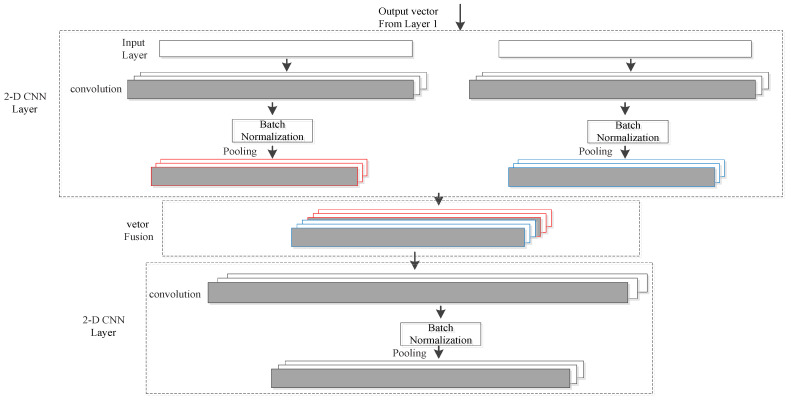
Feature extraction of multi-channel sensor signals.

After the fusion of the feature data stream extracted from each sensor, the fusion features of each sensor data are firstly extracted through the 2D-CNN network. Then, the feature data extracted from multiple sensors are further fused, and the relevant features of each sensor are extracted again through the 2D-CNN network, as shown in Figure 3.

##### 2D-CNN Layer

For the fusion data of each sensor, the one-dimensional time data stream is first converted to the two-dimensional time data stream, and the 2D convolution kernel is used for convolution in the two-dimensional space. Multiple convolution kernels are set between the convolution layers, and multiple feature mappings are learned in the feature map of the previous layer. Let $C_{j}^{l}$ represent the *j*th feature map of the l layer, then

(14) $$C_{j}^{l+1}\left(\tau \right)=\sigma \left(b_{j}^{l}+\overset{F^{l}}{\underset{f=1}{\sum }}K_{jf}^{l}\left(\tau \right)*C_{f}^{l}\left(\tau \right)\right)=\sigma \left(b_{j}^{l}+\overset{F^{l}}{\underset{f=1}{\sum }}\underset{m\epsilon S_{l}}{\sum }K_{jf}^{l}\left(m\right)C_{f}^{l}\left(\tau -m\right)\right)$$

where, σ is the nonlinear activation function, $F^{l}$ represents the number of feature maps at the l layer, $K_{jf}^{l}$ is the convolution kernel of the f-th feature map in layer l to create the j-th feature map in layer l+1, $S_{l}$ represents the feature map set in layer l, and $b^{l}$ is the offset vector.

In the process of model training, in order to reduce the internal covariate shift, a batch normalization layer is set behind each activation layer [26]. We selected the continuous range of feature mapping as the pooling area, and set the max-pooling layer with a size of 2 × 2.

##### Fusion Layer

The human activity data of each sensor are correlated. In order to obtain the correlation features among the sensor activity data and extract the sensitivity fusion features of human activity, the fusion layer was used to fuse the sensor features.

(15) $$C=\left[C^{1}⊚C^{2}\ldots C^{N}\right]$$

where, $C^{i}$ represents the output of the *i*-th sensor fusion feature through the 2D-CNN layer, *C* represents the matrix after the fusion of each sensor feature, and ⊚ represents the splicing of the matrix.

### 3.4. Loss Function

In the process of model training, the ultimate goal of training is to minimize the difference between the predicted labels and the actual labels. In general, cross-entropy loss is used to measure the correlation between labels, as shown in the following formula. The point with the minimum loss is the point with the maximum correlation between the predicted labels and the real labels.

(16) $$loss=-\sum_{i=1}^{M}y_{i}\log\left(p_{i}\right)$$

where, M represents the number of categories, y represents the one-hot vector, $y_{i}$ is 1 if the predicted label and the real label are identical, otherwise, it is 0. The output p of the model is a vector with length M, $p_{i}$ represents the probability of predicting the real label i.

When the samples of classes are unbalanced, the trained model will be biased towards the classes with more samples, leading to the wrong classification of a few sample classes. Therefore, in this paper, focal loss is used as a loss function to reduce the impact of sample imbalance in the hierarchical ensemble deep learning activity recognition model.

(17) $$loss=\overset{M}{\underset{i=1}{\sum }}y_{i}\alpha_{i}{\left(1-p_{i}\right)}^{\gamma_{i}}\log\left(p_{i}\right)$$

where, the hyper-parameter $\alpha_{i}$ represents the equilibrium factor of class i, and the hyper-parameter $\gamma_{i}$ represents the adjustable focusing parameter, which can adjust degree of reduction in the weight of easily classified samples. The greater the $\gamma_{i}$ is, the greater the reduction degree of the weight will be.

In the process of the activity recognition, we collect data from multiple wearable sensors and propose a novel hierarchical ensemble of neural networks, which apply a focal loss algorithm to the activity recognition system for sample imbalance scenarios. The model can reduce the influence of sample imbalance. The training processing of hierarchical ensemble of neural networks model is described in Algorithm 1.
**Algorithm 1** Hierarchical ensemble deep learning model based on focal loss **input**: raw wearable sensor data ** output**: activities 1: encode the raw data as a numeric vector; 2: wavelet transform; 3: normalize the numeric vector; 4: /* Model training*/ 5: **while** the loss does not converge **do**   forward propagation;   use Softmax to get predicted labels;   calculate the focal-loss loss-function;   backpropagation;   gradient descent updates all parameters;   **end**

## 4. Experiment

In this section, experimental settings, data collection and analysis of experimental results are introduced. Tensorflow and Keras Python DL libraries were mainly used to realize the algorithm. The specific settings and results are as follows.

### 4.1. Experimental Settings

#### 4.1.1. The Overview of Experiments

In order to verify the effectiveness of the proposed approach HAR-FL, we designed two types of experiments. One experiment used a dataset that we recruited elderly volunteers to collect, and the other experiment used a public dataset [27].

We took HAR-CE as the benchmark in the following experiments. There is only one difference between HAR-FL and HAR-CE, and that is the adopted loss function, i.e., the loss function adopted by our proposed method HAR-FL was focal loss and the loss function of HAR-CE was cross-entropy loss.

#### 4.1.2. Neural Networks Models

For the two approaches described in Section 4.1.1, we used the same structure of a deep ensemble neural networks model. Specifically, we set the learning rate as $2\times 10^{-4}$ and set batch_size as 128 and used the Adam optimizer. The layers and parameters are shown in Table 2 and Table 3.

Table 2 shows the layers and parameter settings of the first part of the deep ensemble neural networks model, which is named Layer1. In the experiment, each sensor was divided into nine channels, and each channel was set with 1 LSTM layer, 1D-CNN layer, a batch normalization layer (BN layer) and a maximum pooling layer. Then the nine channels were inputted into the fusion layer. All channel features of each sensor were fused.

In this paper, we collected data from two sensors, the features of each sensor fusion were inputted into the 2D-CNN, BN layer and maximum pool. We set up the fusion layer. All the sensor features of the fusion were then inputted into the 2D-CNN layer, BN layer and maximum pool, and extract feature of the sensor fusion. The layers and parameter settings are as shown in Table 3.

Table 4 shows the layers and parameter settings of the regression layer in the third part of the model, which contained five dense layers and a dropouts layer. The output layer was obtained from the Softmax layer (a dense layer with Softmax activation function).

### 4.2. Data Collection and Processing

As discussed in Section 1, our goal in this paper is to identify the daily activities of the elderly to support the monitoring of their health. In order to verify the performance of the activity recognition approach, we recruited ten elderly people in the community, and equipped them with our wearable sensors to collect daily activity data. In the future, we will continue to recruit more elderly people for data collection to further expand our dataset. Elderly volunteers were armed with attitude sensors (model number: BWT61CL), on both wrists and collected the raw sensor data of seven different actions including cooking, playing the keyboard, reading a book, brushing their teeth, washing their face, washing the dishes, writing, etc.

Actually, there are many sensors available for recognizing human activities, such as the attitude sensor, triaxial accelerometer and gyroscope sensor. The differences among them are listed in Table 5.

As shown in Table 4, with one attitude sensor (BWT61CL), we can collect the data of the 3-axis acceleration, 3-axis angular velocity (gyroscope) and 3-axis angle sensors simultaneously. Therefore, we selected the attitude sensor for our work.

Specifically, the model number of the attitude sensor used in our work is BWT61CL and the manufacturer is WitMotion Shenzhen Co., Ltd. (Shenzhen, China). According to the product introduction from the manufacturer’s official website (https://wit-motion.cn, accessed on 31 July 2022), the accuracy of the attitude sensor is guaranteed by the sensor manufacturer’s research and development facilities, e.g., all finished items were calibrated through the world’s top-level triaxial nonmagnetic turntable, ensuring the X Y Z angle’s accuracy. Each sensor of the original data contains nine dimensions (acceleration: 3D, angular velocity: 3D, angle: 3D). When volunteers perform some activities, the sensor on the wrist can receive data in real-time through the host computer. The data collection time for each action is about 7 min.

The specific data collection and processing process was as follows:

Prior to inputting the training datasets into the model, we set each sensor data window size as 50, the step size of the sliding window was 25, and we divided the sensor data flow for the same size. Each sample was a matrix, whose size was 50 (about 5 s) × 2 (motion sensor) × 9 (nine-axis sensor data). The data, which were reconstructed into the required time-series format of the model, were used as the input of the hierarchical ensemble neural network model.

Table 6 shows classes imbalance, where S1 represents the benchmark case with uniformly distributed classes, S2–S5 denotes four cases, in which the number of samples in two classes is 200, and the number of samples in the rest classes is 708.

### 4.3. Analysis of Experimental Results

#### 4.3.1. Analysis of Experimental Results with Private Dataset

**Figure 4:**
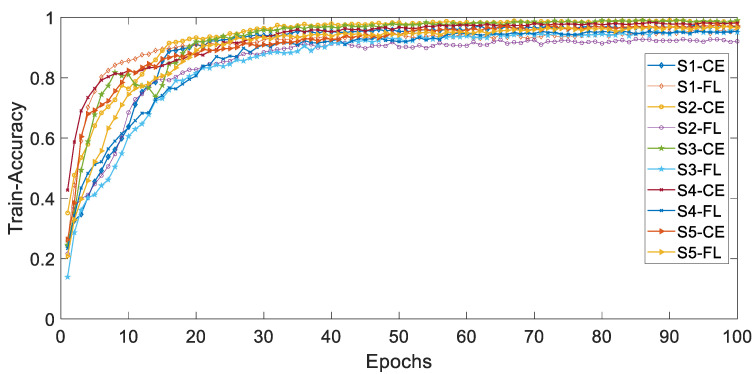
The curve of accuracy with training set.

**Figure 5:**
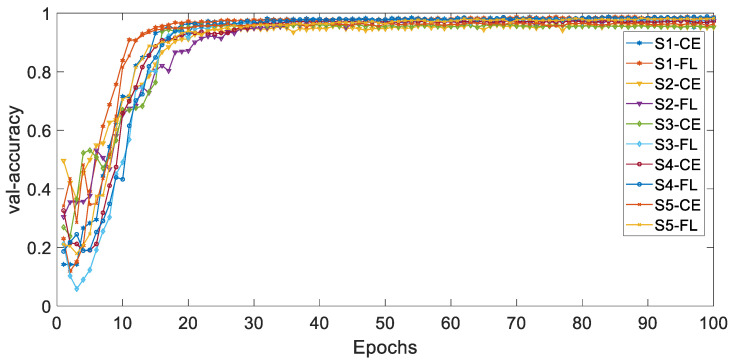
The curve of accuracy with validation set.

Figure 4 and Figure 5, respectively, show the curves of the accuracy of training and validation sets with epochs under different types of imbalances based on different loss functions. The adjustable focusing parameter γ is 2, and the balance weight α is 0.25. It can be seen from the curves of the two figures that, with the epochs getting larger, the overall trend of train-accuracy and val accuracy tends to 1. The convergence rate of the training set accuracy of HAR-CE is faster than that of HAR-FL. Except for S1, the convergence rate of the accuracy of HAR-CE with validation set is faster than that of HAR-FL.

Table 7 shows the results of the precision, recall and F1-score of HAR-CE and HAR-FL under different classes of imbalance. It can be seen from the results in the table that HAR-FL is significantly better than HAR-CE, and the experimental results under balanced data classes are better than those under unbalanced data classes.

**Figure 6:**
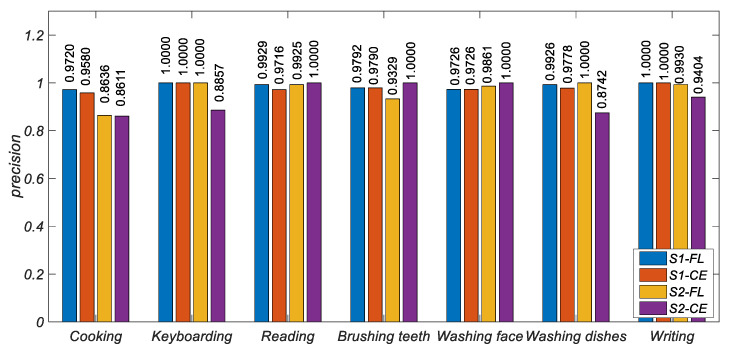
The precision comparison of HAR-CE and HAR-FL in each class.

**Figure 7:**
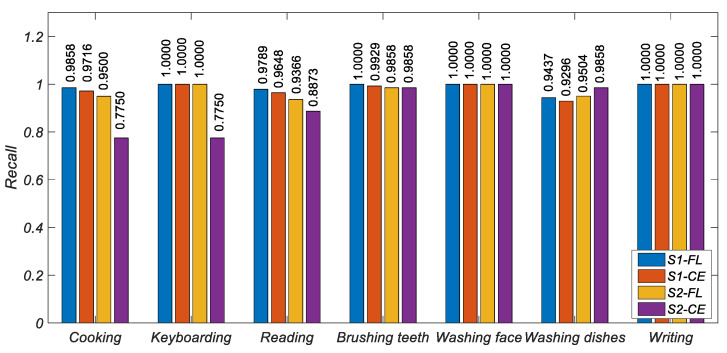
The recall comparison of HAR-CE and HAR-FL in each class.

**Figure 8:**
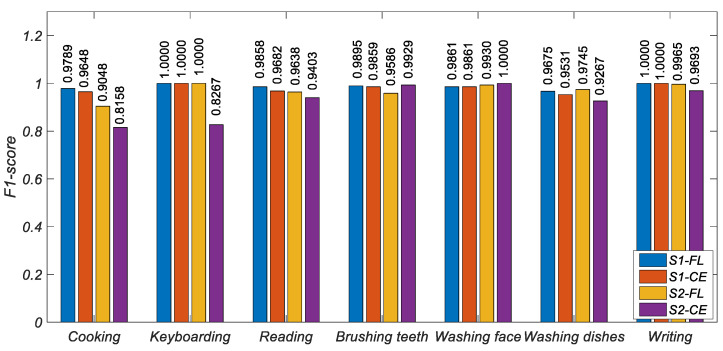
The F1-score comparison of HAR-CE and HAR-FL in each class.

Figure 6, Figure 7 and Figure 8 show the histogram of the results of various metrics of HAR-CE and HAR-FL. It can be seen from the figure that the results of various metrics of HAR-FL of most classes are better than those of HAR-CE except for some classes. In the case of balanced S1, the difference between HAR-CE and HAR-FL is small. In the case of unbalanced S2, HAR-FL is significantly better than HAR-CE.

**Figure 9:**
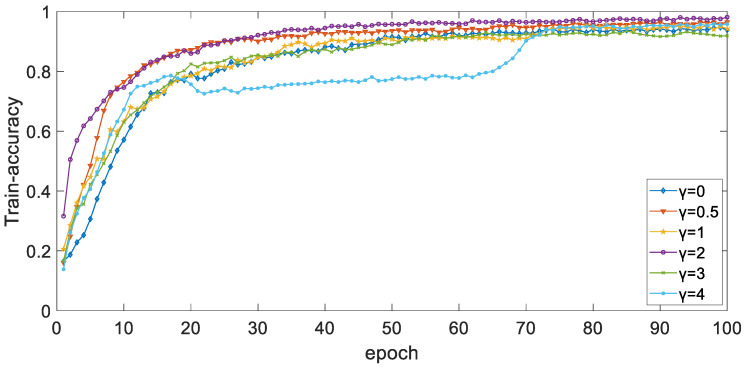
The curve of accuracy of training set with different epoch when parameter γ is different.

**Figure 10:**
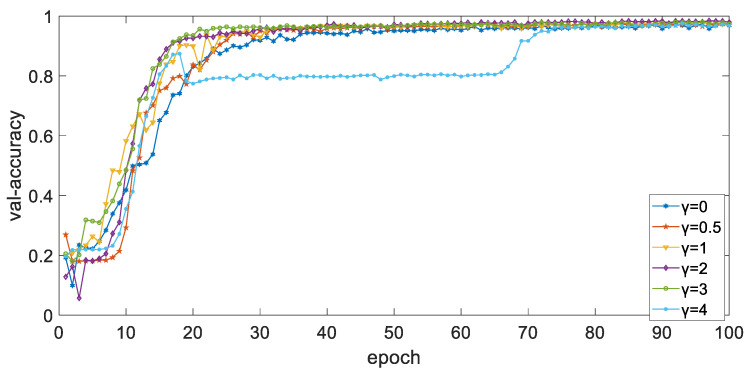
The curve of accuracy of validation set with different epoch when parameter γ is different.

By adjusting the γ value in the focal loss function, the weight of easily classic samples and hard classic samples in the loss function can be dynamically adjusted, so that the model can focus more on hard classic samples. In the case of S2, the balance weight α is set to 0.25, the learning rate is $2\times 10^{-4}$, and the Adam optimizer is used. When batch_size is set to 128, Figure 9 and Figure 10 show the curve of accuracy of training set and validation set with a different epoch when parameter γ is different. It can be seen from the figure that the overall trend of accuracy of the training set and validation set tends to 1 as the epoch increases, and the performance when γ is 2 is better than that when γ is other values.

**Figure 11:**
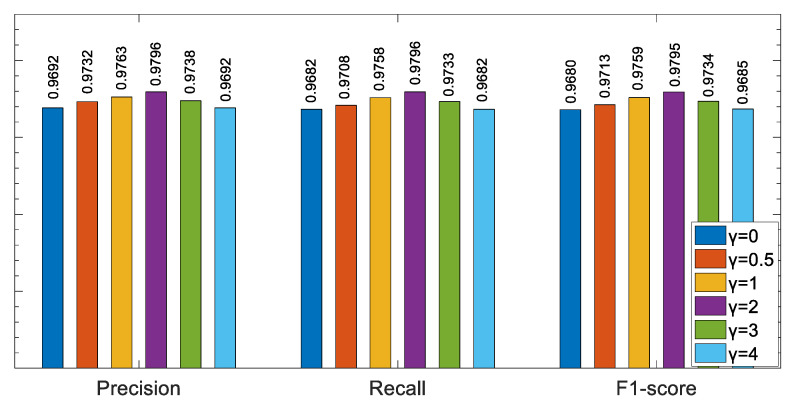
The comparison of model performance under different γ.

Precision, recall and the F1-score were used to evaluate the model performance under different values of γ. The experimental results are shown in Figure 11. It can be seen from the figure that when the parameter γ is 2, the model performance of each metric is significantly better than that when γ is other values.

In summary, when the datasets are class balanced, there is little difference between the performance of each metric of HAR-CE and HAR-FL. In the case of class imbalance, the performance of HAR-FL is significantly better than that of HAR-CE. When the balance weight α is 0.25 and γ is 2, the performance of HAR-FL is the best.

#### 4.3.2. Analysis of Experimental Results with Public Dataset

In order to comprehensively evaluate the performance of our proposed approach, the heterogeneous dataset (D_H_) [27] was used to verify the model. The D_H_ dataset is composed of the data which was collected by eight smartphones for six daily activities (‘Biking‘, ‘Sitting’, ‘Standing’, ‘Walking’, ‘StairsUp’, ‘StairsDown’) of nine users. The original data contains six dimensions (accelerometer: 3D, gyroscope: 3D). To ensure consistency, they collected each activity data for 5 min. The specific dataset attributes are shown in Table 8.

In this paper, we selected from data DH that collected by users “b” and “e” carrying four mobile phones: “NexUS4_1”, “NexUS4_2”, “S3mini_1” and “S3mini_2”. Table 9 shows the table of class distribution. S1 represents the benchmark situation containing uniformly distributed classes, S2 represents the situation when the activity is “upstairs”; the number of the active data windows is 2560. S3 represents the distribution that the number of the active data windows is 2559 when the activity is “riding a bike”.

**Figure 12:**
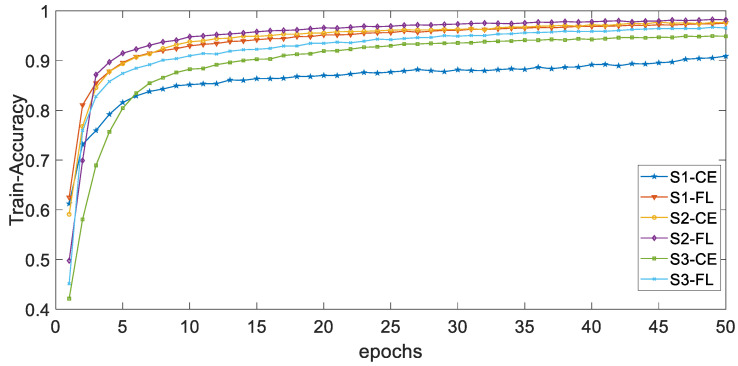
The curves of the accuracy of DH training sets with epochs.

**Figure 13:**
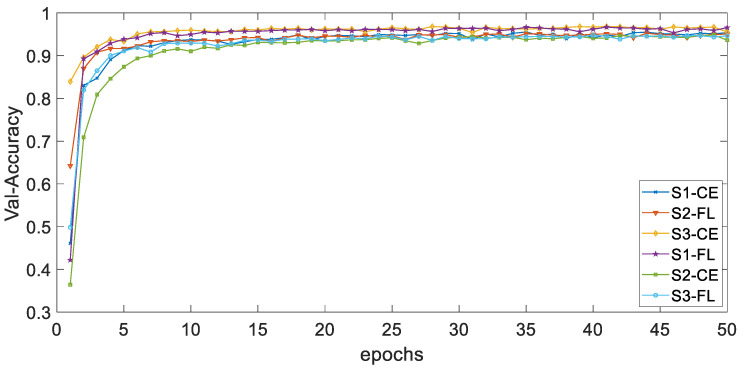
The curves of the accuracy of D_H_ validation sets with epochs.

Figure 12 and Figure 13 respectively show the curves of the accuracy of training and validation sets with epochs under different classes numbers of distribution of dataset D_H_. The adjustable focusing parameter γ is 2, and the balance weight α is 0.25. It can be seen from the curves of the two figures that, with the increase of epochs, the overall trend of train-accuracy and val accuracy tends to a stable value. When epochs are in the range of 0–5, the convergence rate is fast; when epochs are in the range of 5–50, the convergence rate is slow and tends to be stable.

Table 10 shows the results of the accuracy, recall, and F1-score of HAR-CE and HAR-FL under different classes of imbalance. It can be seen from the results in the table that HAR-FL is significantly better than HAR-CE. When the sample distribution is S2, the indicators of S2 outperformed the other distributions mainly due to a reduction in the number of classes that are more difficult to classify.

Table 11 presents the results of various indicators of HAR-CE and HAR-FL. It can be seen from the table that the results of various indicators of HAR-FL in most classes are better than those of HAR-CE. When the class is “Stairsup” or “Stairsdown”, each performance indicator is significantly lower than the other classes. It can be determined that the data with activities of “upstairs” or “downstairs” have high similarity.

## 5. Conclusions

It is particularly important to recognize the daily activities of the elderly, which can be further used to predict and monitor chronic diseases, such as dementia. According to the unbalanced features of data classes, in this paper we propose a hierarchical ensemble deep learning activity recognition approach with wearable sensors based on focal loss. In our approach, wearable sensor devices are worn on both wrists to collect a variety of human daily activity data.

The experimental results show that, for the activity data with imbalanced classes, the hierarchical ensemble deep learning model based on focal loss has a good effect in recognition activity.

The daily activities of the elderly have different effects on sensors worn on different parts. Therefore, how to balance the influence factors of each sensor on activities is the focus of our future work.

## Figures and Tables

**Table 1:** The comparison of related work.

Reference	Main Contributions	Sensor	Classes
[7]	The real-time activity recognition application on a smartphone with the Google Android platform	smartphone	stand, walk, stair up/down, run, shopping, taking bus, moving (by walk)
[8]	The activity recognition model permits users to gain useful knowledge about the habits of millions of users passively just by having them carry cell phones	smartphone	walking, jogging, climbing stairs, sitting, and standing
[12]	Proposed a deep convolutional neural network (convnet) is to perform HAR using smartphone sensors by exploiting the inherent characteristics of activities and 1D time-series signals	smartphone	walking, upstairs, downstairs, sitting and standing, lying
[14]	Evaluating what is the best descriptor to recognize human activity using Convolutional Neural Network in a non-controlled environment using a network of smart objects	smartphone	standing, sitting, lying and walking
[15]	Developed modified training procedures for LSTM networks and combine sets of diverse LSTM learners into classifier collectives	wearable sensors	close/open dishwasher, close/open drawer, close/open door, close/open fridge, toggle switch, drink from cup, clean table
[17]	Investigating the opportunity to use deep learning to perform this integration of sensor data from multiple sensors	smartphone	sitting, standing, walking, climbing stairs, descending stairs, biking
[18]	Proposed a generic deep framework for activity recognition based on convolutional and LSTM recurrent units	wearable sensors	close/open dishwasher, close/open drawer, close/open door, close/open fridge, toggle switch, drink from cup, clean table
[19]	Introduced a novel ensemble ELM algorithm for human activity recognition using smartphone sensors	smartphone	sitting, standing, lying, walking, walking upstairs, and downstairs

**Table 2:** Layer 1 construction.

Layers	#Feature Maps	Feature Map Size	#Parameters
LSTM	32	28	4352
1D-CNN	8	28	1288
BN	8	28	32
Max-pooling1D	8	14	0
Concatenate	72	14	0
Reshape	1	14 × 72	0

**Table 3:** Layer 2 construction.

Layers	#Feature Maps	Feature Map Size	#Parameters
2D-CNN	8	14 × 72	80
BN	8	14 × 72	32
Max-pooling2D	8	7 × 36	0
Concatenate	16	7 × 36	0
2D-CNN	32	7 × 36	4640
BN	32	7 × 36	128
Max-pooling2D	32	4 × 18	0

**Table 4:** Regression layer construction.

Layers	#Feature Maps	Feature Map Size	#Parameters
Flatten	1	2304	0
Dense-1	1	64	147,520
Dense-2	1	32	2080
Dense-3	1	16	528
Dropout	1	16	0
Dense-4	1	8	136
Dense-5	1	7	63

**Table 5:** The comparison of different sensors.

Sensors	Attitude Sensor (BWT61CL)	Triaxial Accelerometer	Gyroscope Sensor
3-Axis Acceleration	✓	✓	
3-Axis Angular Velocity (Gyroscope)	✓		✓
3-Axis Angle	✓		

**Table 6:** Classes distribution.

Classes	S1	S2	S3	S4	S5
Cooking	708	200	708	708	200
Keyboarding	708	200	708	708	708
Reading	708	708	200	708	708
Brushing teeth	708	708	200	708	708
Washing face	708	708	708	200	708
Washing dishes	708	708	708	200	708
Writing	708	708	708	708	200

**Table 7:** Comparison of HAR-CE and HAR-FL.

Samples	HAR-FL			HAR-CE		
	Precision	Recall	F1-Score	Precision	Recall	F1-Score
S1	0.9870	0.9869	0.9868	0.9799	0.9798	0.9797
S2	0.9759	0.9746	0.9747	0.9539	0.9517	0.9511
S3	0.9723	0.9720	0.9715	0.9549	0.9543	0.9534
S4	0.9850	0.9847	0.9846	0.9729	0.9720	0.9710
S5	0.9823	0.9822	0.9821	0.9636	0.9632	0.9625

**Table 8:** Heterogeneity dataset (D_H_) characterized by their respective attributes.

Activities	Devices	FS	Users
[”Biking”, ”Sitting”, ”Walking”, ”StairsUp”, “StairsDown”, “Standing”]	Nexus 4	200	[a,b,c,d,e,f,g,h,i]
	Samsung S3	150	
	Samsung S3 Mini	100	
	Samsung S+	50	

**Table 9:** The class distribution of D_H_ dataset.

	Classes	S1	S2	S3
Class Distribution	stand	7932	7932	7932
	sit	8089	8089	8089
	walk	10,225	10,224	10,225
	stairsup	7519	2560	7519
	stairsdown	6607	6607	6607
	bike	9580	9580	2559

**Table 10:** The comparison results of HAR-CE and HAR-FL with D_H_ data set.

Samples	HAR-FL			HAR-CE		
	Precision	Recall	F1-Score	Precision	Recall	F1-Score
S1	0.9640	0.9641	0.9640	0.9569	0.9571	0.9570
S2	0.9720	0.9717	0.9718	0.9660	0.9656	0.9657
S3	0.9474	0.9465	0.9466	0.9388	0.9362	0.9363

**Table 11:** The distribution of indicators for each class of HAR-FL and HAR-CE with D_H_ dataset.

Classes	HAR-FL			HAR-CE		
	Precision	Recall	F1-Score	Precision	Recall	F1-Score
stand	0.9950	0.9965	0.9970	0.9965	0.9960	0.9962
sit	1.000	0.9990	0.9997	1.0000	0.9995	0.9998
walk	0.9626	0.9660	0.9590	0.9618	0.9460	0.9588
stairsup	0.9089	0.9128	0.9067	0.9008	0.9021	0.9055
stairsdown	0.8777	0.8660	0.8783	0.8747	0.8644	0.8710
bike	0.9858	0.9880	0.9894	0.9744	0.9871	0.9864

## Data Availability

The data that support the findings of this study are available from the corresponding author upon reasonable request.

## References

[1] Xie B., Wu Q. Hmm-based tri-training algorithm in human activity recognition with smartphone. Proceedings of the 2012 IEEE 2nd International Conference on Cloud Computing and Intelligence Systems.

[2] Sarcevic P., Kincses Z., Pletl S. Comparison of different classifiers in movement recognition using WSN-based wrist-mounted sensors. Proceedings of the 2015 IEEE Sensors Applications Symposium (SAS).

[3] Fan L., Wang Z., Wang H. Human activity recognition model based on decision tree. Proceedings of the 2013 International Conference on Advanced Cloud and Big Data.

[4] He K., Zhang X., Ren S., Sun J. Deep residual learning for image recognition. Proceedings of the IEEE Conference on Computer Vision and Pattern Recognition.

[5] Donahue J., Anne Hendricks L., Guadarrama S., Rohrbach M., Venugopalan S., Saenko K., Darrell T. Long-term recurrent convolutional networks for visual recognition and description. Proceedings of the IEEE Conference on Computer Vision and Pattern Recognition.

[6] Lane N.D., Georgiev P. Can deep learning revolutionize mobile sensing?. Proceedings of the 16th International Workshop on Mobile Computing Systems and Applications.

[7] Lee Y.S., Cho S.B. Activity recognition using hierarchical hidden markov models on a smartphone with 3D accelerometer. Proceedings of the International Conference on Hybrid Artificial Intelligence Systems.

[8] Kwapisz J.R., Weiss G.M., Moore S.A. (2011). Activity recognition using cell phone accelerometers. ACM Sigkdd Explor. Newsl..

[9] Sun L., Zhang D., Li B., Guo B., Li S. Activity recognition on an accelerometer embedded mobile phone with varying positions and orientations. Proceedings of the International Conference on Ubiquitous Intelligence and Computing.

[10] LeCun Y., Bengio Y., Hinton G. (2015). Deep learning. Nature.

[11] Jiang W., Yin Z. Human activity recognition using wearable sensors by deep convolutional neural networks. Proceedings of the 23rd ACM International Conference on Multimedia.

[12] Ronao C.A., Cho S.B. (2016). Human activity recognition with smartphone sensors using deep learning neural networks. Expert Syst. Appl..

[13] Ravi D., Wong C., Lo B., Yang G.Z. Deep learning for human activity recognition: A resource efficient implementation on low-power devices. Proceedings of the 2016 IEEE 13th International Conference on Wearable and Implantable Body Sensor Networks (BSN).

[14] Amroun H., Temkit M.H., Ammi M. Best feature for CNN classification of human activity using IOT network. Proceedings of the 2017 IEEE International Conference on Internet of Things (iThings) and IEEE Green Computing and Communications (GreenCom) and IEEE Cyber, Physical and Social Computing (CPSCom) and IEEE Smart Data (SmartData).

[15] Guan Y., Plötz T. Ensembles of deep lstm learners for activity recognition using wearables. Proceedings of the ACM on Interactive, Mobile, Wearable and Ubiquitous Technologies.

[16] Kuncheva L.I. (2014). Combining Pattern Classifiers: Methods and Algorithms.

[17] Radu V., Lane N.D., Bhattacharya S., Mascolo C., Marina M.K., Kawsar F. Towards multimodal deep learning for activity recognition on mobile devices. Proceedings of the 2016 ACM International Joint Conference on Pervasive and Ubiquitous Computing.

[18] Ordóñez F.J., Roggen D. (2016). Deep convolutional and lstm recurrent neural networks for multimodal wearable activity recognition. Sensors.

[19] Chen Z., Jiang C., Xie L. (2018). A novel ensemble ELM for human activity recognition using smartphone sensors. IEEE Trans. Ind. Inform..

[20] Sundaramoorthy P., Gudur G.K., Moorthy M.R., Bhandari R.N., Vijayaraghavan V. HARNet: Towards on-device incremental learning using deep ensembles on constrained devices. Proceedings of the 2nd International Workshop on Embedded and Mobile Deep Learning.

[21] Wang K.J., Makond B., Chen K.H., Wang K.M. (2014). A hybrid classifier combining SMOTE with PSO to estimate 5-year survivability of breast cancer patients. Appl. Soft Comput..

[22] Jo T., Japkowicz N. (2004). Class imbalances versus small disjuncts. ACM Sigkdd Explor. Newsl..

[23] Hradsky O., Ohem J., Zarubova K., Mitrova K., Durilova M., Kotalova R., Nevoral J., Zemanova I., Dryak P., Bronsky J. (2014). Disease activity is an important factor for indeterminate interferon-γ release assay results in children with inflammatory bowel disease. J. Pediatr. Gastroenterol. Nutr..

[24] Lin T.Y., Goyal P., Girshick R., He K., Dollár P. Focal loss for dense object detection. Proceedings of the IEEE International Conference on Computer Vision.

[25] Borovykh A., Bohte S., Oosterlee C.W. (2017). Conditional time series forecasting with convolutional neural networks. arXiv.

[26] Ioffe S., Szegedy C. Batch normalization: Accelerating deep network training by reducing internal covariate shift. Proceedings of the International Conference on Machine Learning.

[27] Stisen A., Blunck H., Bhattacharya S., Prentow T.S., Kjærgaard M.B., Dey A., Sonne T., Jensen M.M. Smart devices are different: Assessing and mitigatingmobile sensing heterogeneities for activity recognition. Proceedings of the 13th ACM Conference on Embedded Networked Sensor Systems.

